# PDGF Suppresses Oxidative Stress Induced Ca^2+^ Overload and Calpain Activation in Neurons

**DOI:** 10.1155/2013/367206

**Published:** 2013-12-24

**Authors:** Lian-Shun Zheng, Yoko Ishii, Qing-Li Zhao, Takashi Kondo, Masakiyo Sasahara

**Affiliations:** ^1^Institute of Anatomy and Cell Biology, School of Medicine, Zhejiang University, Hangzhou, China; ^2^Department of Pathology, Graduate School of Medicine and Pharmaceutical Sciences for Research, University of Toyama, 2630 Sugitani Toyama, Toyama 930-0194, Japan; ^3^Department of Radiological Sciences, Graduate School of Medicine and Pharmaceutical Sciences for Research, University of Toyama, Toyama, Japan

## Abstract

Oxidative stress is crucially involved in the pathogenesis of neurological diseases such as stroke and degenerative diseases. We previously demonstrated that platelet-derived growth factors (PDGFs) protected neurons from H_2_O_2_-induced oxidative stress and indicated the involvement of PI3K-Akt and MAP kinases as an underlying mechanism. Ca^2+^ overload has been shown to mediate the neurotoxic effects of oxidative stress and excitotoxicity. We examined the effects of PDGFs on H_2_O_2_-induced Ca^2+^ overload in primary cultured neurons to further clarify their neuroprotective mechanism. H_2_O_2_-induced Ca^2+^ overload in neurons in a dose-dependent manner, while pretreating neurons with PDGF-BB for 24 hours largely suppressed it. In a comparative study, the suppressive effects of PDGF-BB were more potent than those of PDGF-AA. We then evaluated calpain activation, which was induced by Ca^2+^ overload and mediated both apoptotic and nonapoptotic cell death. H_2_O_2_-induced calpain activation in neurons in a dose-dependent manner. Pretreatment of PDGF-BB completely blocked H_2_O_2_-induced calpain activation. To the best of our knowledge, the present study is the first to demonstrate the mechanism underlying the neuroprotective effects of PDGF against oxidative stress via the suppression of Ca^2+^ overload and inactivation of calpain and suggests that PDGF-BB may be a potential therapeutic target of neurological diseases.

## 1. Introduction

Oxidative stress and excitotoxicity play important roles in the pathogenesis of a number of neurological diseases, including ischemic infarction, multiple sclerosis, amyotrophic lateral sclerosis, and Alzheimer's, Huntington's, and Parkinson's diseases [1–3]. Ca^2+^ has been shown to mediate the cytotoxicity of oxidative stress and excitotoxicity, and cellular Ca^2+^ overload or the perturbation of intracellular Ca^2+^ compartmentalization induced by these noxious stimuli can cause cytotoxicity and trigger cell death including both apoptotic and necrotic cell death [4–6]; however, these mechanisms of cellular injury have yet to be elucidated in adequate detail to prevent and treat neurological diseases [7, 8].

Calpains are calcium-regulated cysteine proteases that have been implicated in the regulation of cell death pathways including apoptosis and necrosis [9, 10]. An elevated intracellular calcium concentration will hyperactivate calpains. The activation of calpains was shown to be involved in various pathological conditions, including ischemic brain injuries and chronic neurodegenerative diseases, for example, Alzheimer's disease [9, 11]. Previous studies reported that calpain inhibitors were neuroprotective in free radical injury models associated with mitochondrial dysfunction [12], apoptotic injury following spinal cord trauma [13], and traumatic brain injury [14]. Neural degeneration and apoptosis were shown to be ameliorated in calpain-1 null mice following traumatic brain injury [15]. Therefore, suppressing Ca^2+^ overload and the activation of calpain are a crucial strategy to overcome neurological diseases mediated by oxidative stress and excitotoxicity.

The platelet-derived growth factor (PDGF) family members, PDGF-A, -B, -C, and -D, are assembled as disulfide-linked homo- or heterodimers, and two receptor tyrosine kinases, PDGFR-*α* and -*β*, which can form homo- and heterodimeric receptor complexes, have been identified [16]. PDGFR-*αα* was previously shown to be activated by PDGF-AA, -AB, -CC, and -BB, PDGFR-*αβ* by PDGF-AB, -BB, and -CC, and PDGFR-*ββ* by PDGF-BB and -DD.

Previous studies demonstrated that PDGF and PDGFRs were widely expressed in the central nervous system (CNS) [17–19]. A neuroprotective role has been hypothesized based on the findings of a number of studies; either the suppression of PDGF-B or conditional deletion of the PDGFR-*β* gene resulted in the enhanced vulnerability of the CNS to excitotoxicity or ischemia [20–22]. Furthermore, our recent studies demonstrated that PDGF-AA and -BB protected cultured neurons against oxidative stress and suppressed H_2_O_2_-induced caspase-3 activation through PDGFR-*α* or -*β* expressed on these cells [23]. In this study, PI3-K/Akt and MAP kinase pathways were suggested to mediate neuroprotective effects. PDGF-CC was reported to exert neuroprotective effects through the activation of GSK3beta both *in vivo* and *vitro* [24]. However, the neuroprotective mechanism underlying PDGFR signaling has not yet been clarified.

We herein identified another neuroprotective pathway mediated by PDGFs. PDGF-AA and PDGF-BB suppressed the Ca^2+^ overload induced by H_2_O_2_ in primary cultured mouse cortical neurons. Furthermore, PDGF-BB attenuated the H_2_O_2_-induced activation of calpain, which is one of the key molecules of neuronal dysfunction induced by oxidative stress and Ca^2+^ overload [10]. Therefore, this study provides a novel insight into the mechanism underlying the neuroprotective effects of PDGF against oxidative stress.

## 2. Experimental Procedures

### 2.1. Mice

We used wild-type C57BL/6J mice (Sankyo Laboratory, Toyama, Japan). Mice were maintained with free access to laboratory pellet chow and water and exposed to a 12 h light/12 h dark cycle. All animal procedures were performed according to the Institutional Animal Care and Use Committee Guidelines at the University of Toyama under an approved protocol.

### 2.2. Cell Cultures

Cell cultures were established as previously mentioned [23]. Briefly, cerebral cortices were dissected from neonatal mice on postnatal day 1, enzymatically dissociated in 0.1% trypsin (Nacalai Tesque, Kyoto, Japan) for 5 min at 37°C, and were then mechanically dissociated with fire-polished Pasteur pipettes. Following centrifugation (150 ×g for 5 min), cells were resuspended in Dulbecco's modified Eagle's medium (DMEM)/F12 medium (1 : 1; Invitrogen, Carlsbad, CA) containing 10% fetal bovine serum (FBS; HyClone, Yokohama, Japan) and were maintained in serum-free neurobasal medium supplemented with 1% B27 supplement (Invitrogen), 2 mM L-glutamine (Sigma, Louis, MO), 100 units/mL penicillin (Invitrogen), and 0.1 mg/mL streptomycin (Invitrogen). Cells were then plated on glass-bottomed culture dishes (P35G-0-10-C, MatTek, Ashland, MA) at a density of 4.2 × 10^4^ cells/cm^2^ to determine the intracellular concentration of the calcium ion ([Ca^2+^]_*i*_). To determine calpain activity, cells were plated on 24-well plates (BD Biosciences, San Jose, CA) at a density of 1 × 10^5^ cells/cm^2^. All dishes and plates were precoated with 0.001% poly-L-lysine (Sigma). Fresh medium was added every 3 days and cultures were maintained. Fewer than 5% of cultured cells were glia because more than 95% were MAP-2-positive neurons with morphologically mature features, such as extending neurites, at 7 days *in vitro* (DIV).

### 2.3. Drug Treatments

Recombinant human PDGF-AA and PDGF-BB were purchased from Chemicon (Temecula, CA). Oxidative stress was induced by a treatment with H_2_O_2_ for 24 h at DIV 7 as previously described [23]. To investigate the effects of PDGF on H_2_O_2_-induced [Ca^2+^]_*i*_, neurons were pretreated with PDGF for 24 h. After loading Fura-2-AM (Dojindo, Kumamoto, Japan), cells were transferred into fresh media containing H_2_O_2_. PDGF was not included in this fresh medium in order to avoid the acute effects of freshly provided PDGF on [Ca^2+^]_*i*_. To determine the effects of PDGF on H_2_O_2_-induced calpain activity, neurons pretreated with PDGF for 24 or 48 h were exposed to H_2_O_2_ prepared in media containing PDGF for 24 h and were then processed to determine calpain activity.

### 2.4. Ca^**2+**^ Imaging Analysis: Determination of the Intracellular Concentration of Calcium Ions

[Ca^2+^]_*i*_ was evaluated as described elsewhere [25, 26]. Briefly, 1 *μ*M Fura-2-AM (Dojindo) solution was prepared using loading buffer, which was HEPES-buffered Ringer solution supplemented with 0.2% bovine serum albumin (Sigma), Eagle's minimal essential amino acids (Flow Laboratories, Surrey UK), and 2 mM L-glutamine. HEPES-buffered Ringer solution (pH 7.4) contained 118 mM NaCl, 4.7 mM KCl, 2.5 mM CaCl_2_, 1.13 mM MgCl_2_, 1 mM Na_2_HPO_4_, 5.5 mM glucose, and 10 mM HEPES-KOH. After the 24 h PDGF pretreatment, cells were washed with PBS and loaded with 1 *μ*M Fura-2-AM solution for 15 min at room temperature (25°C). Cells were washed twice with PBS, which was then replaced with cultured media supplemented with or without H_2_O_2_ for up to 30 min. Digital images of Fura-2 fluorescence were acquired and analyzed by a digital image processor (Argus 50/CA, Hamamatsu Photonics, Hamamatsu, Japan) coupled with an inverted fluorescent microscope [25]. The ratio of 510 nm emission fluorescence at 340 nm excitation to that at 380 nm excitation, F (340/380), was used as an indicator of [Ca^2+^]_*i*_ in cortical neurons. Pseudocolor images of individual cells and mean F (340/380) values were obtained 15 and 30 min after the treatment with H_2_O_2_.

### 2.5. Calpain Activity Assay

Activated calpain released into the cytosol was extracted, and the activities of calpain-1 and -2 were determined using the Calpain Activity Assay kit (Biovision, Milpitas, CA) according to the manufacturer's instruction. Briefly, cultured neurons were incubated with lysis buffer for 20 min at 4°C. Clarified cell lysates after centrifugation were incubated with reaction buffer containing a substrate of calpain (Ac-LLY-AFC) for 1 h at 37°C in the dark. Upon cleavage of the substrate, the fluorogenic portion (7-amino-4-trifluoromethyl coumarin) yielded 505 nm fluorescence emission at 400 nm excitation. Fluorescence emission was measured by a standard fluorimeter (FilterMax F5, Molecular Devices, Sunnyvale, CA). Control reactions were performed for each sample in the presence of an inhibitor of calpain-1 and -2 to monitor any calpain-independent proteolysis of the fluorogenic peptide. Values from control reactions were subtracted from total activity values to specifically determine calpain activity for each sample. Results are expressed as relative fluorescence units per milligram of lysate protein.

## 3. Statistical Analysis

Quantitative data were expressed as means ± SEM, and each experiment was repeated at least three times. A one-way ANOVA followed by Fisher's PLSD test used for statistical analysis, with *P* values less than 0.05 was being considered significant.

## 4. Results

### 4.1. PDGF-BB Attenuated the H_**2**_O_**2**_-Induced Increase in the Intracellular Calcium Ion Concentration

**Figure 1:**
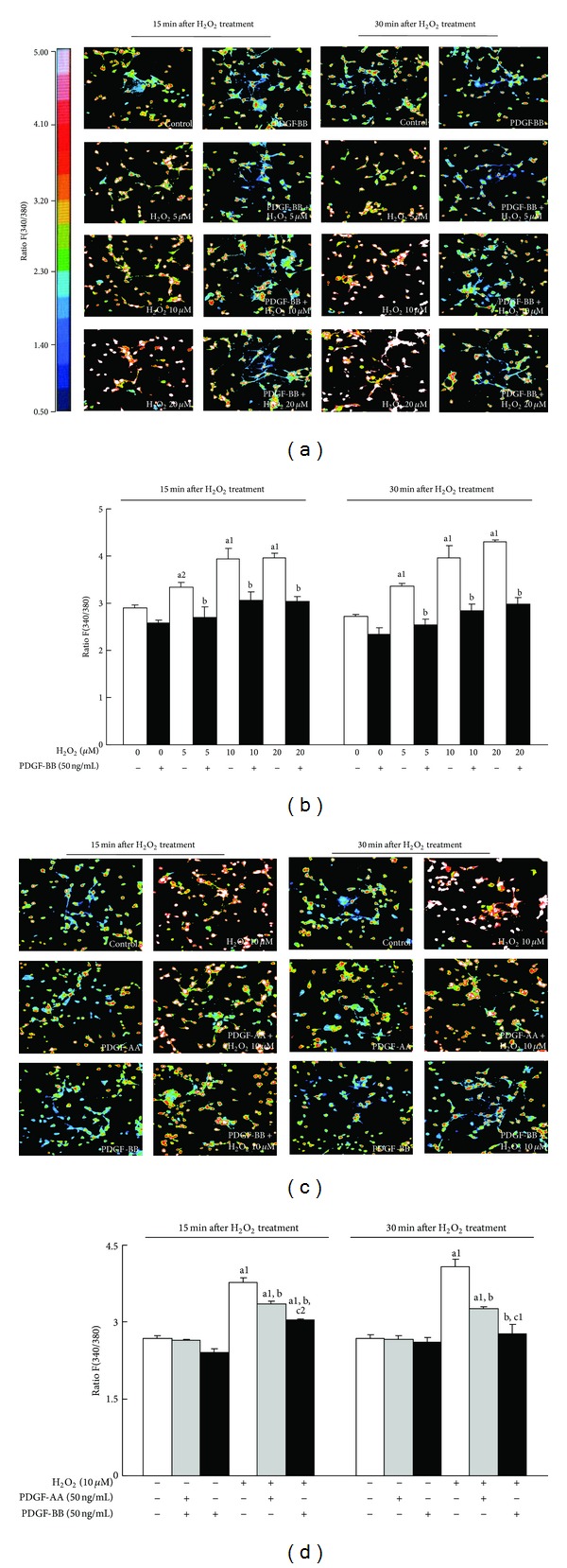
Effects of PDGF-AA and PDGF-BB on the H_2_O_2_-induced increase in [Ca^2+^]_*i*_. [Ca^2+^]_*i*_ was determined using Fura-2-AM fluorescent dye. (a) Pseudocolor images representing the relative [Ca^2+^]_*i*_ indicated by the fluorescence ratio between 340 and 380 nm (F340/380) in individual cortical neurons 15 and 30 min after the H_2_O_2_ treatment. Following a 24 h preincubation with 50 ng/mL PDGF-BB, neurons were loaded with Fura-2-AM and exposed to different concentrations of H_2_O_2_ (5, 10, and 20 *μ*M). The inserted bar indicates the relationship between colors and fluorescent intensity ratios at 340 and 380 nm. (b) Histogram analyses of the mean F340/380 of (a). Three pictures were taken from each well, and the means of the F340/380 of each neuron were calculated. Data are expressed as means ± SEM derived from three different sets of experiments. (c) To compare the effects of PDGF-AA and PDGF-BB on [Ca^2+^]_*i*_ overload induced by H_2_O_2_, cells were pretreated with 50 ng/mL PDGF-AA or PDGF-BB for 24 h followed by exposure to 10 *μ*M H_2_O_2_ for 15 and 30 min. (d) Histogram analysis of (c). Data are expressed as means ± SEM of three independent experiments. ^a1^ *P* < 0.01 and ^a2^ *P* < 0.05 versus the untreated control; ^b^ *P* < 0.01 versus the same H_2_O_2_ exposure without the PDGF pretreatment; ^c1^ *P* < 0.01 and ^c2^ *P* < 0.05 versus the same H_2_O_2_ exposure with the PDGF-AA pretreatment.

The neuroprotective effects of PDGFs against H_2_O_2_ have been reported previously [23]; therefore, we examined the effects of PDGFs on the H_2_O_2_-induced overload of [Ca^2+^]_*i*_, which has been implicated in oxidative stress-induced cellular injury [27, 28]. On *in situ* pseudocolor images, control neurons that were not exposed to H_2_O_2_ frequently showed low [Ca^2+^]_*i*_, and many neurons showed high [Ca^2+^]_*i*_ after H_2_O_2_ at 15 and 30 min (Figure 1(a)). The number of neurons showing high [Ca^2+^]_*i*_ after H_2_O_2_ appeared to be decreased by the 24 h pretreatment with PDGF-BB at both 15 and 30 min (Figure 1(a)). The means of [Ca^2+^]_*i*_ evaluated from these images demonstrated that the PDGF-BB pretreatment did not affect [Ca^2+^]_*i*_ in the control neurons without H_2_O_2_ exposure (Figure 1(b)). The H_2_O_2_ treatment increased [Ca^2+^]_*i*_ in neurons in a dose-dependent manner up to 5 and 20 *μ*M at 15 and 30 min, respectively, (Figure 1(b)). This H_2_O_2_-induced [Ca^2+^]_*i*_ overload was completely abolished by the PDGF-BB pretreatment under all conditions examined.

We then compared the effect of PDGF-AA and -BB on [Ca^2+^]_*i*_ overload after the H_2_O_2_ treatment. On *in situ* pseudo-color images of relative [Ca^2+^]_*i*_, many neurons showed high [Ca^2+^]_*i*_ after the 10 *μ*M H_2_O_2_ treatment (Figure 1(c)). Either the PDGF-AA or PDGF-BB pretreatment appeared to decrease the number of neurons showing high [Ca^2+^]_*i*_ after 10 *μ*M H_2_O_2_ (Figure 1(c)). Analyses of the mean [Ca^2+^]_*i*_ indicated that either the PDGF-AA or PDGF-BB pretreatment did not affect [Ca^2+^]_*i*_ in the control neurons without H_2_O_2_ treatment (Figure 1(d)). The H_2_O_2_ treatment significantly induced [Ca^2+^]_*i*_ overload at 15 and 30 min. PDGF-AA significantly inhibited this [Ca^2+^]_*i*_ overload. This inhibition was partial, and [Ca^2+^]_*i*_ after H_2_O_2_ in neurons pretreated with PDGF-AA was significantly higher than that in control neurons without the H_2_O_2_ treatment. [Ca^2+^]_*i*_ in neurons pretreated with PDGF-BB was significantly lower than that in neurons pretreated with PDGF-AA at 15 and 30 min and was similar to that in the controls at 30 min.

### 4.2. PDGF-BB Attenuated the H_**2**_O_**2**_-Induced Increase in Active Calpain

**Figure 2:**
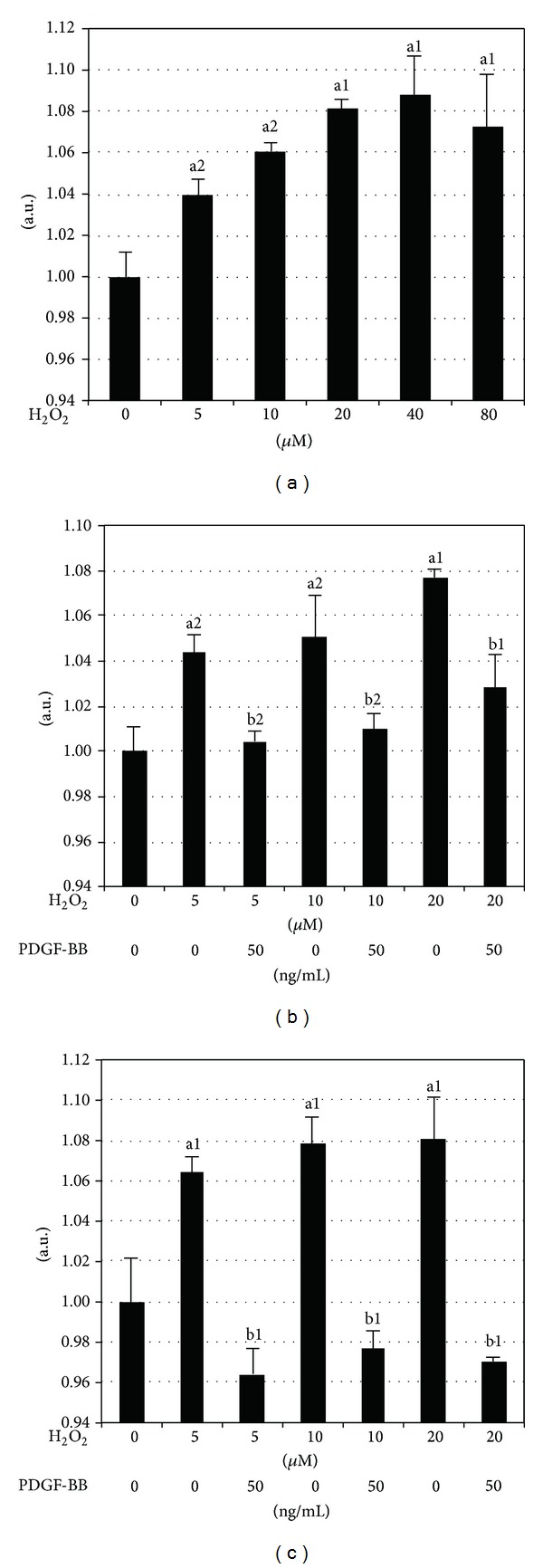
Effects of PDGF-BB on H_2_O_2_-induced calpain activation. Calpain-1 activity was measured 24 h after exposure to H_2_O_2_ as described in the Experimental procedures. (a) Calpain activity induced by the indicated amounts of H_2_O_2_ (5, 10, 20, 40, and 80 *μ*M) exposure increased calpain activation in cortical neurons in a dose-dependent manner from 5–20 *μ*M. ((b), (c)) Calpain activity in neuronal cultures pretreated with 50 ng/mL PDGF-BB 24 h (b) and 48 h (c) before exposure to H_2_O_2_ PDGF-BB completely blocked calpain activity induced by H_2_O_2_. Data are expressed as means ± SEM of three independent experiments. ^a1^ *P* < 0.01 and ^a2^ *P* < 0.05 versus the untreated control; ^b1^ *P* < 0.01 and ^b2^ *P* < 0.05 versus the same H_2_O_2_ exposure without the PDGF-BB pretreatment.

Because the PDGF pretreatment suppressed H_2_O_2_-induced [Ca^2+^]_*i*_ overload, we examined whether PDGF suppressed calpain activation, which is a downstream mediator of [Ca^2+^]_*i*_ overload that induces cellular injury. We determined the activities of calpain-1 and -2, as these were shown to be the major subtypes of the calpain family that mediate neurological diseases [9]. The H_2_O_2_ treatment activated calpain in cultured neurons in a dose-dependent manner from 5 *μ*M to 20 *μ*M, and their activities remained high to similar extents from 20 *μ*M to 80 *μ*M of H_2_O_2_ (Figure 2(a)). H_2_O_2_-induced calpain activation in neurons pretreated for 24 h with PDGF-BB significantly decreased from 5 to 20 *μ*M of H_2_O_2_ to a similar level as that in neurons without the H_2_O_2_ treatment (Figure 2(b)). Although H_2_O_2_-induced calpain activation in neurons pretreated for 48 h with PDGF-BB appeared to be decreased to lower levels than the control, this difference was not significant (Figure 2(c)).

## 5. Discussion

In the present study, we examined a PDGF-mediated neuroprotective pathway against H_2_O_2_-induced oxidative stress. Increased cytosolic Ca^2+^ and subsequent calpain activation represent one of the major pathways underlying reactive oxidative species (ROS)-mediated cell death [9]. In the present study, the H_2_O_2_-induced Ca^2+^ increase and calpain activation in cultured neurons were markedly suppressed by PDGF and were suggested to be the targets of a neuroprotective mechanism by PDGF.

The oxidative stress-induced Ca^2+^ overload in cultured neurons was markedly suppressed by PDGF-BB and, to a lesser extent, by PDGF-AA. The oxidative stress-induced inward Ca^2+^ current has been shown to trigger several downstream lethal reactions, including nitrosative and oxidative stress, mitochondrial dysfunction, and protease and phospholipase activation, which culminate in cell death [5, 28]. This Ca^2+^-pathway may be one of the central mechanisms underlying the death of neurons subjected to ischemia and energy deprivation. The Ca^2+^ chelator BAPTA/AM was shown to induce a decrease in intracellular Ca^2+^ and almost completely blocked H_2_O_2_-induced apoptosis [29]. Thus, the inhibition of Ca^2+^ overload may be one mechanism underlying PDGF-mediated neuroprotection [30], and this mechanism could correspond, at least partly, to the PDGF-induced suppression of neuronal cell death exposed to H_2_O_2_ [23]. A previous study demonstrated that NGF and bFGF protected cultured hippocampal neurons by suppressing increases in Ca^2+^ due to glucose deprivation, which was consistent with our results [31].

In the present study, PDGF-AA significantly suppressed H_2_O_2_-induced Ca^2+^ overload. PDGF-BB suppressed Ca^2+^ overload more potently than PDGF-AA. PDGF-BB was previously shown to activate two types of PDGFRs to high levels, while PDGF-AA activated PDGFR-*α*, but not PDGFR-*β* in cultured neurons [23]. Accordingly, two types of PDGFR were suggested to mediate the suppressive effects of Ca^2+^ overload, respectively, and the additive effects of the two activated PDGFRs may explain the more potent effects of PDGF-BB than those of PDGF-AA. Alternatively, distinctive signaling downstream of these two PDGFRs may account for the different effects of PDGF-AA and -BB; for example, PDGFR-*β* was shown to potently activate the PI3-Akt pathway, whereas it activated the MAP kinase pathway to a similar extent to that of PDGFR-*α*, as demonstrated in a PDGFR-*β* knockout study in cultured neurons [23].

Calpain has been shown to be activated by either ROS or NMDA-induced Ca^2+^ overload [32]. Calpain 1 (*μ*-calpain) and calpain 2 (m-calpain) exist as a proenzyme heterodimer (80 kDa–29 kDa) in resting cells, and they are activated by Ca^2+^ in autolytic processing (to produce a heterodimer 78 kDa–18 kDa) [9, 10]. This activated calpain further disturbs mitochondrial Ca^2+^ metabolism and plays a pivotal role in inducing distinctive types of cell death including apoptosis, necrosis, and autophagy [9, 10, 33]; for example, calpain-1 mediated the cleavage of autophagy-related gene 5, which is a critical switch from protective autophagy to cell death in the presence of apoptotic stimuli [33]. In our previous study conducted in the same experimental condition as present study, PDGF-BB suppressed both apoptotic and nonapoptotic cell death induced by H_2_O_2_ [23]. Accordingly, these findings indicate that the suppressive effects of PDGF on calpain activity may correspond to the neuroprotective effects of PDGF including apoptotic and non-apoptotic prosurvival mechanisms.

Evidence is accumulating to show that Ca^2+^ overload and the activation of calpain mediate excitotoxic neuronal injury [9, 34–36]. PDGF-B protected primary cultured neurons from NMDA-induced excitotoxicity [37]. We reported that the suppression of PDGF-B mRNA expression by antisense oligonucleotides exaggerated NMDA-induced excitotoxicity in neonatal rat brains [20] and that adult mouse brains that expressed reduced levels of neuronal PDGFR-*β* had more lesions after NMDA-induced excitotoxicity or cryogenic injury [21]. Accordingly, the effects of PDGF on Ca^2+^ overload and calpain activation shown in the present study may correspond to the underlying mechanism of PDGF to suppress excitotoxicity. An inward Ca^2+^ current after oxidative stress was shown to be evoked through NMDA receptors and transient receptor potential (TRP) channels, which belong to a group of ion channels [1, 38]. PDGF suppressed the inward Ca^2+^ current through NMDA receptors [39, 40], which may be involved in the antiexcitotoxic effect of PDGF; however, further studies are required to clarify the effects of PDGF on neuronal cell metabolism [30].

A previous report demonstrated that PDGF-AA and PDGF-BB protected hippocampal neurons subjected to glucose deprivation or exposed to the hydroxyl radical-promoting agent, FeSO_4_, due to the induction of antioxidant enzymes [41]. The activation of Akt and MAP kinase was shown to mediate prosurvival effects in neurons exposed to H_2_O_2_-induced oxidative stress [23]. PDGF-CC exerted neuroprotective effects via the activation of GSK3beta [24]. Therefore, the presently reported effects on Ca^2+^ and calpain metabolism were suggested to be a novel neuroprotective mechanism of PDGF. Calpain and Ca^2+^ elevations have been shown to mediate both acute and chronic cell death, such as ischemic/traumatic brain injuries and Alzheimer's disease, respectively [9, 10]. Our studies identified PDGF as a potential therapeutic intervention in neurons exposed to oxidative stress. Further studies are needed to investigate the role of PDGF-BB in the pathway of neuronal death induced by oxidative stress.

PDGF-BB is one of the intrinsic neurotrophic factors abundantly expressed in the brain and is upregulated in response to brain insults [17, 42]. In parallel to the on-going clinical phase I/II trial of PDGF-BB in Parkinson's patients [43], further basic studies are required to find out the effective therapeutic strategies targeting PDGF-BB.
